# FSH regulates acetycholine production by ovarian granulosa cells

**DOI:** 10.1186/1477-7827-4-37

**Published:** 2006-07-17

**Authors:** Artur Mayerhofer, Lars Kunz, Annette Krieger, Becky Proskocil, Eliot Spindel, Abraham Amsterdam, Gregory A Dissen, Sergio R Ojeda, Ignaz Wessler

**Affiliations:** 1 Anatomisches Institut der Universität München, Deutschland; 2 Division of Neurosciences, ONPRC-OHSU, Beaverton, OR, USA; 3 Weizmann Institute of Science, Department of Molecular and Cellular Biology, Rehovot, Israel; 4 Phamakologisches Institut der Universität Mainz, Deutschland

## Abstract

**Background:**

It has been previously shown that cultured granulosa cells (GCs) derived from human ovarian preovulatory follicles contain choline acetyltransferase (ChAT), the enzyme responsible for acetylcholine (ACh) synthesis. They also produce ACh and express functional muscarinic ACh receptors. ACh can act on GCs to increase proliferation, disrupt gap junctional communication, alter intracellular calcium levels, as well as expression of transcription factors, suggesting an unrecognized role of ACh in GC function. To gain further insights into the possible role of ACh in the ovary, we examined ChAT expression in the gland before and after birth, as well as in adults, and studied the regulation of ACh production by FSH.

**Methods:**

ChAT immunohistochemistry was performed using ovarian samples of different species and ages (embryonic, postnatal and adult rats and mice, including embryonic ovaries from mice null for ChAT, neonatal and adult rhesus monkeys and adult humans). ACh was measured by HPLC and/or a fluorescence based method in rat ovaries and in a FSH receptor-expressing cell line (rat GFSHR-17) cultured with or without FSH.

**Results:**

In adult rat, as well as in all other species, ovarian ChAT immunoreactivity is associated with GCs of antral follicles, but not with other structures, indicating that GCs are the only ovarian source of ACh. Indeed ACh was clearly detected in adult rat ovaries by two methods. ChAT immunoreactivity is absent from embryonic and/or neonatal ovaries (mouse/rat and monkey) and ovarian development in embryonic mice null for ChAT appears normal, suggesting that ACh is not involved in ovarian or follicular formation. Since ChAT immunoreactivity is present in GCs of large follicles and since the degree of the ChAT immunoreactivity increases as antral follicles grow, we tested whether ACh production is stimulated by FSH. Rat GFSHR-17 cells that stably express the FSH receptor, respond to FSH with an increase in ACh production.

**Conclusion:**

ACh and ChAT are present in GCs of growing follicles and FSH, the major driving force of follicular growth, stimulates ACh production. Since ACh stimulates proliferation and differentiation processes in cultured GCs, we suggest that ACh may act in the growing ovarian follicle as a local mediator of some of the actions ascribed to FSH.

## Introduction

Several publications have challenged the concept that acetycholine (ACh) in the ovary derives from a cholinergic innervation of the gland [see [1-5]]. Instead, ACh is produced by non-neuronal cells of the ovary, and specifically by granulosa cells (GCs). HPLC measurements provided unequivocal evidence for ACh production by human GCs derived from large preovulatory follicles of women undergoing in vitro fertilization, and for ACh production in a rat cell line (GFSHR-17) [6], derived from an antral rat follicle (see summary in [1]).

Furthermore, ChAT protein was detected in GCs of large antral follicles by immunohistochemisty [3,7-9]. These morphological observations, together with a host of data showing actions of cholinergic agents on cultured human GCs [7-11] provide evidence for the concept that ACh is produced locally in the ovary, and acts within the gland to affect specific ovarian functions (see also [1-3]). Thus, human GCs contain muscarinic ACh receptors (MR) and ACh regulates cell proliferation via MR, inhibits gap-juctional communication, and among other actions, stimulates the transcription factor egr-1 (early growth response factor 1) [9]. MR of human GCs are G-protein coupled receptors linked to increases in intracellular calcium levels. They are also linked to activation of calcium activated potassium channels (BK_Ca _[11]). Data from this study also indicate that activated BK_Ca _channels are necessary for hCG to exert its stimulatory action on steroid production [11]. It is thus possible that an intraovarian ACh/MR signaling complex may be complementary to the cAMP/PKA signaling pathway used by gonadotrophins in GCs [12,13]. Relevant to this issue is the demonstration that oocytes possess functional MRs and represent, therefore, also a target for ACh [8,14,15].

Whether ACh production is restricted to preovulatory follicles and how the production of ACh by antral follicles is regulated, remain unknown [16]. We have, therefore, examined the ovarian sites of ChAT expression in rodents and primates and have determined whether ovarian morphology is altered in ChAT knockout mice. We also measured ACh levels in adult ovaries and tested the possibility that FSH, the hormone required for antral follicular growth, may affect ACh production by GCs.

## Methods

### Tissues

Paraffin-embedded, fixed human ovaries (n = 3) were from patients undergoing gynecological surgery. The procedures were approved by the local Ethics Committees and patients gave written consent to the use of tissues. Additional samples (n = 4) were from a tissue bank, as described [17]. Sections (10 μm) were cut and used for immunohistochemistry.

Monkey ovarian samples (obtained at necropsy or during surgery for unrelated reasons at the ONPRC-OHSU) were from postnatal day 0, 1, and 3–17 years of age, total of 10 samples). The care and housing of rhesus macaces (Macaca mulatta) at the ONPRC was previously described [11,17]. Animal protocols and experiments were approved by the ONPRC Animal Care and Use Committee, and studies were conducted in accordance with the NIH Guide for the Care and Use of Laboratory Animals. Sections (10 μm) were cut from all paraffin-embedded samples and were used for immunohistochemistry.

Mouse and rat ovarian samples used (from embryonic stages, i.e. post coitus (p.c.) 17, 18, 20, postnatal day 0, day 1 and adult) were derived from previous studies [7,18-20].

ChAT knockout (-/-) mice were generously provided by Fred Gage and Kuo-Fen Lee [21]. Mice were genotyped as described [21] using primers ATGATGACAGGCAACAAGAAGC, CTTCCATTTGTCACGTCCTGC and AGGGATGAGAATGGGTGGAGCC, which resulted in PCR products of 240 bp for the wildtype gene and 364 bp for the knockout gene, respectively. Heterozygous males and females were mated and the day of conception was determined by presence of a vaginal plug or sperm in the vagina. Fetuses were obtained by caesarian section on day 18 p.c. and ovaries dissected out. Fetuses were genotyped from tail DNA and ovaries from ChAT (-/-) and wildtype control mice (+/+) were fixed in Bouin's and embedded in paraffin. Sections (10 μm) of 5 ovaries of each group were made and were stained with H.E.

### Immunohistochemistry and immunofluorescence

The ABC method and DAB as a chromogen were used for immunohistochemistry, while FITC-labeled secondary antiserum was used for immunofluorescence. All procedures were performed as described [9,11,20,22]. A specific mouse anti ChAT monoclonal antibody (1:500 to 1:1,000; Boehringer Mannheim, Mannheim, Germany) was used. For controls, incubation with mouse normal serum, IgG or buffer without the specific antibody was employed, as described. Incubations with the primary antibody were carried out overnight at 4°C, incubations with the secondary antiserum were done at room temperature for 2 h.

### ACh measurement in rat ovaries

We studied whether ACh is present in adult rat ovaries. To this end ovaries from 11 adult rats, used in unrelated experiments, were examined. Animals were kept at the animal house of the Technical University of Munich and killed according to NIH/local animal care guidelines.

Ovaries were rapidly removed, frozen on liquid nitrogen and ACh was either determined using the HPLC method, as described previously (n = 6 adults; see [7,8]) or a fluorescence-based method (5 adults). To perform the latter, each ovary was homogenized in 500 μl cold 10 mM PBS on ice and samples were either stored at -80°C until use or were used immediately. Before measurement they were centrifuged and the supernatant was subjected to ACh detection using the Amplex Red Acetylcholine Assay kit (Molecular Probes/Invitrogen GmbH, Karlsruhe, Germany).

Briefly, in this assay ACh is hydrolyzed by ACh esterase (AChE), the choline formed is then oxidized by choline oxidase, and the H_2_O_2 _resulting from this reaction, interacts with Amplex Red (7-dihydroxyphenoxazine) in the presence of horseradish peroxidase to form the highly fluorescent resorufin. The samples were transferred into a 96-well plate and measured in a fluorescence plate reader (BMG Labtech GmbH, Offenburg, Germany) every 5 min for 40 min. All measurements were performed in triplicates. As this assay detects not only ACh, but also choline, we measured each sample in the absence of AChE, as well. Sensitivity of the assay was as low as 0.3 μM, with a range of detection from 0.3 μM to 100 μM ACh. For quantification purposes, ACh solutions in the range of 0 μM to 3 μM were measured, and a calibration curve was generated from the values obtained. The ratio of fluorescence intensities obtained in the presence of AChE divided by those in its absence was 1.2 ± 0.1 (n = 5 ovaries from 5 animals; mean ± SD; one-sample t-test, P = 0.0014). A value larger than one indicates that ACh was present in the samples. The choline detected by the assay can derive from endogenous sources and/or originate from non-enzymatic hydrolysis of ACh and/or from enzymatic hydrolysis of ACh by intrinsic esterase activity of the tissue. Thus, to semi-quantitatively assess ACh concentrations we subtracted the choline values obtained in absence of AChE from those in its presence.

### Culture of GFSHR-17 cells for ACh determination

GFSHR-17 cells are derived from antral rat follicles, but are not able to produce estrogens [23]. Yet, they respond to FSH [6,23]. All methods, including description of culture conditions have been reported in detail [23]. In brief, cells were cultured with Dulbecco modified Eagle medium Ham's F12 (1:1 (v/v); Biochrom KG, Berlin, Germany) containing 5% fetal calf serum (Biochrom KG, Berlin, Germany). Cells were maintained in culture for up to 25 passages and were treated as indicated with/without porcine (p) FSH (0.5 IU/ml; Sigma, Deissenhofen, Germany) for 24 h and with lower concentrations for pilot studies. As reported previously, pFSH only slightly stimulates progesterone production by GFSHR-17 cells [23]. Cellular content of ACh was determined (8 samples/group from 3 independent experiments) as described previously [7,8,16] and was normalized to cellular protein. Results are expressed as means ± SEM. Statistical significance of changes was determined using t-test.

## Results

### ChAT immunoreactivity in the ovary

**Figure 1:**
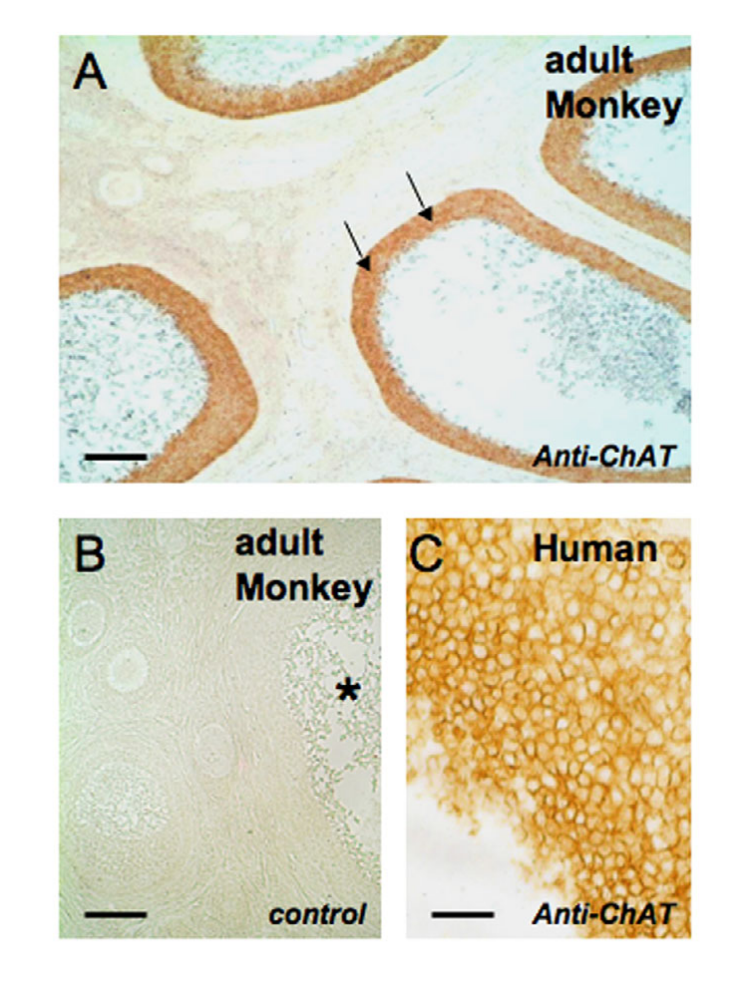
**ChAT in adult primate ovaries**. A: ChAT is detectable using immunohistochemical methods in a monkey ovary only in the GC layer (arrows) of several large antral follicles. Note that the areas between the sectioned follicles are devoid of any immunoreactive structures. Bar = 500 μm. B: A consecutive section of the one shown in A is depicted and used as a control (incubation with non-immune mouse normal serum instead of the specific antibody). The asterisk denotes a large antral follicle. Bar = 200 μm. C. Immunoreactive GCs of a large antral follicle in a human ovary. Bar = 50 μm.

**Figure 2:**
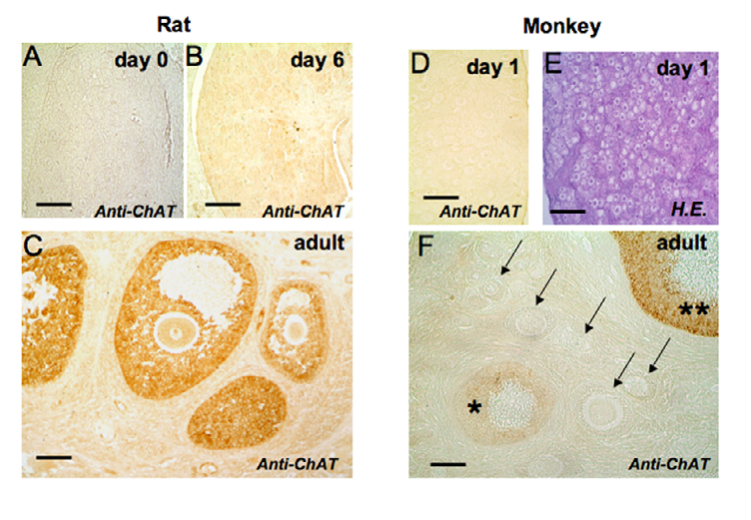
**ChAT is present in growing antral follicles of rat and monkey ovary**. A-C: Rat ovaries: ChAT is not detectable using immunohistochemical methods in a rat ovary at postnatal days 0 or 6 (A, B). Bars = 50 μm. In adult rat ChAT (C) is present in GCs of antral follicles. Bar = 100 μm. D-F: Monkey ovaries: ChAT is not detectable using immunohistochemical methods in a monkey ovary at postnatal day 1 (D). The same ovary is shown after staining with H.E. to reveal structural details of follicle formation (E). Bars = 50 μm. F: Immunohistochemistry of an adult monkey ovary showing a large antral follicle (two asterisks) strongly stained for ChAT, preantral follicles (arrows) devoid of staining, and the appearance of immunoreactivity in follicles with incipient antrum formation (asterisk). Bar = 100 μm.

**Figure 3:**
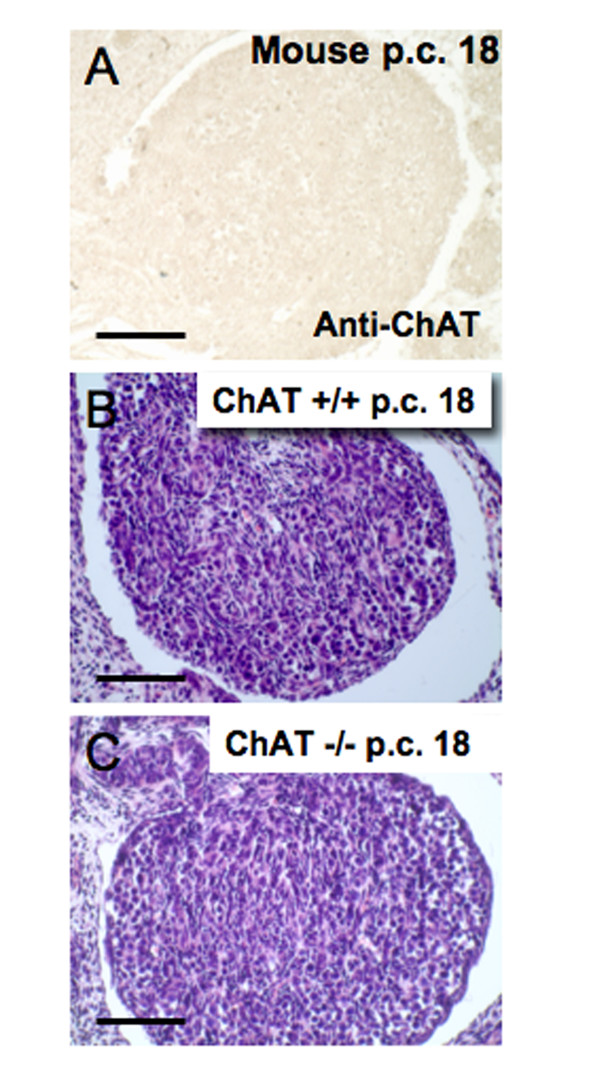
**Absence of ChAT in embryonic mouse ovary and morphology of ChAT (-/-) mouse ovary**. A: ChAT is not detectable using immunohistochemical methods in a mouse ovary at day 18 p.c. Bar = 50 μm. B-C: H.E. stained sections of the ovary of an embryonic (day 18 p.c.) age-matched wild-type ovary (+/+) and a mutant mouse null for ChAT (-/-). Bars = 60 μm.

ChAT immunoreactivity, as expected, was observed in human, monkey and rat adult ovaries only in GCs of large antral follicles, but not in small follicles (see Fig. 1 and 2). Immunoreactive nerve fibers, expected to be present in interstitial or thecal areas and around blood vessels, were not observed. ChAT was absent from neonatal or postnatal (day 0 and 6) rat ovary (Fig. 2). Likewise, neonatal (day 1) monkey ovaries were devoid of ChAT immunoreactivity, as were rat and mouse embryonic (p.c. 18) ovaries (Fig. 2, 3; rat not shown).

Furthermore ovarian development in embryonic mutant mice null for ChAT was normal with respect to organ size and cellular composition, ruling out an involvement of ACh in the early ovarian development (Fig. 3).

### Ovarian ACh levels

Expression of ChAT in adult rat ovaries is associated with the presence of ACh (n = 6); the mean value was 2.47 ± 0.90 pmol (mean ± SEM) as detected by a HPLC technique. The presence of ACh in rat ovaries was corroborated using a fluorescence technique (n = 5), and the values obtained ranged between 22 and 60 pmol (mean ± SEM: 43 ± 7 pmol).

### Regulation of ACh production by FSH in a rat GC line

**Figure 4:**
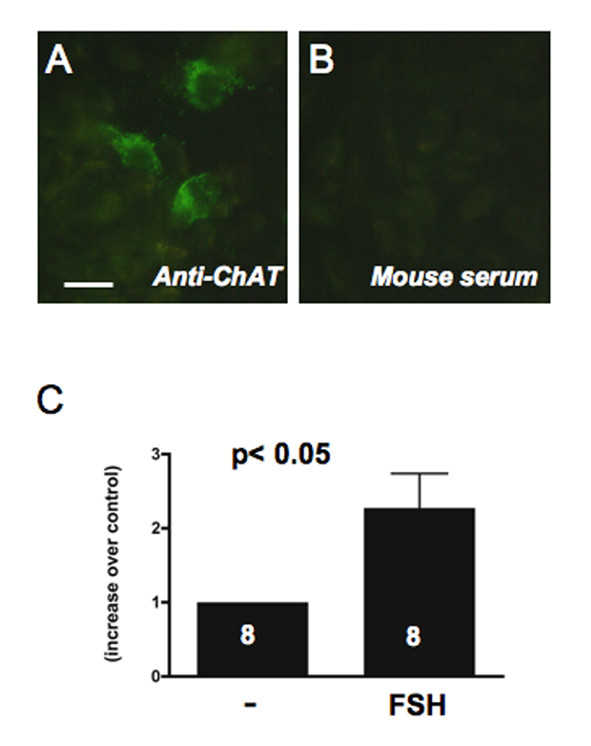
**Regulation of ACh production in GCs by FSH**. A: Immunostaining of ChAT in GFSHR-17 cells and B: lack of staining in control cells incubated with mouse serum instead of the specific antibody. Bar = 10 μm. C: Rat GFSHR-17 cells incubated for 24 h with 0.5 mIU/ml of porcine FSH respond with a significant increase in ACh production. Results shown represent means and SEM of 8 samples per group. Results of untreated cells were set to 1 (t-test; p < 0.05).

Since follicular growth and GC development depend on FSH, we hypothesized that ACh production may be stimulated by the gonadotrophin. To test this possibility we determined the production of ACh in the rat GFSHR-17 cell line challenged with pFSH for 24 h. This cell line stably expresses the FSH receptor and is immunoreactive for ChAT (Fig. 4). Using a HPLC detection system, we found a statistically signifcant increase in ACh production (p < 0.05; Fig. 4C).

## Discussion

The present results demonstrate (1) the presence of ACh in adult rat ovary and (2) indicate that GCs of growing antral ovarian follicles in both rodents and primates are the only detectable structures, which are immunoreactive for ChAT, implying that GCs are the source of ovarian ACh. Furthermore (3) our results indicate that ChAT staining increases in GCs of growing antral ovarian follicles in both rodents and primates and (4) show that FSH stimulates production of ACh in cells derived from an antral follicle. Finally, (5) since ChAT is absent from embryonic and neonatal ovary and embryonic development in ChAT-/- mice appears normal, these data support the hypothesis that ACh is a signaling molecule important for the antral follicle, which is produced by non neuronal cells of the ovary under gonadotrophin control.

A role of ACh in the ovary has only recently been suggested, based on studies with human GCs [1]. These cells, derived from the preovulatory follicle of women undergoing in vitro fertilization procedures, bear resemblance to preovulatory follicular GCs. In the present study we sought to further define this ovarian site of presumed ACh synthesis.

We confirmed that in rodents and primates only growing follicles express ChAT, and thus, are likely to produce ACh. The ChAT protein is not found in embryonic and neonatal ovary and is first detected in follicles that are beginning to develop an antrum, i.e. a stage supported by FSH, which drives subsequent follicular growth [12,13,24]. Since preantral follicles lack ChAT, ACh may not be important for events taking place before antrum formation, including the embryonic phase of ovarian development. Unfortunately, ChAT null mice die at birth [21] precluding examination of postnatal ovarian development.

The increased ChAT immunoreactive signal detected in larger antral follicles, as compared with smaller antral follicles, suggested that ACh production may be regulated by FSH. This conclusion is supported by the results of our studies with immortalized GCs, which stably express functional FSH receptors [23], as these cells responded to FSH with increased production of ACh. This increase likely reflects an enhanced production of ACh per cell, because GFSHR-17 cells do not proliferate when treated with FSH [23], and the ACh values were expressed in relation to cellular protein content. A potential involvement of estrogens in ACh production can be ruled out (see [23]). Although estrogens are also produced by GCs of growing follicles in response to FSH, GFSHR-17 cells do not produce estrogens when challenged with FSH (see [23] for details). These cells however produce small amounts of progesterone [23]. The cellular mechanisms employed by FSH to increase ACh production remain to be elucidated.

We clearly detected ACh itself by two techniques in homogenates from adult rat ovaries, in which ChAT immunoreactive GCs are present. Preliminary unpublished studies with immature rat ovarian homogenates (from ovaries of days 1–6) have sofar indicated that ACh in these samples is below detection limits. These results are consistent with the observed and documented lack of ChAT in the neonatal ovary (present study). More detailed studies, which are beyond the present report, are however required to examine ovarian ACh levels during sexual maturation and correlate them with ovarian and follicular development.

When measuring ovarian ACh content in adults we did not differentiate between ovaries bearing differently sized follicles or corpora lutea and the results obtained reflect a thus expected heterogeneity. Furthermore the two different methods employed provided somewhat different absolute values. Several reasons may account for these differences, which have to do with the principles of the assays used (HPLC detection versus enzymatic assay). Furthermore, it is possible that the magnitude of the changes is, at least in part, determined by changes in ACh metabolism. ACh is a very labile molecule with a very short half life time. Our preliminary data from fluorescence ACh assays strongly suggests presence of esterases degrading ACh, and preliminary Western blot data implicates butyrylcholine esterase [25,26] as one of the esterases involved. Clearly, additional studies are required to elucidate the mechanisms of inactivation of ACh by GCs in the ovary.

ACh has only recently been recognized as a "cyto-transmitter" that, produced by non-neuronal cells, can act throughout the body in an autocrine and/or paracrine fashion (see [27]). It is now known that a variety of cells, including epithelial cells, endothelial cells, cancer cells, immune cells and placental tissue produce ACh and possess ACh receptors [16,28,29]. Importantly, ChAT activity of human placental cells is not affected by LH or FSH [28], suggesting that an FSH-dependent regulation in GCs is a cell-specific event.

## Conclusion

ACh and ChAT are present in GCs of growing follicles and the hormone FSH, the major driving force of follicular growth, stimulates ACh production. Since ACh stimulates proliferation and differentiation processes in cultured GCs (see summary in [1-3]), we suggest that ACh may act in the growing ovarian follicle as a local mediator of some of the actions ascribed to FSH.

## Declaration of competing interests

The author(s) declare that they have no competing interests.

## Authors' contributions

AM conceived of the study, coordinated the work, analyzed the data, and drafted the manuscript. BP and ES were responsible for breeding and genotyping ChAT null and wild-type mice and dissecting of the ovaries. They also contributed to the writing of the paper. AA provided GFSHR-17 cells and contributed to the manuscript. GAD and SRO collected monkey ovaries and made major contributions to writing of the manuscript. AK was involved in cell culture experiments and together with LK developed and performed all fluorescence ACh measurements, and both were involved in drafting of the mansucript. IW performed all HPLC ACh measurements, helped in analysis of the data and writing of the paper. All authors read and approved of the manuscript.
